# Anti-tax interacting protein-1 (TIP-1) monoclonal antibody targets human cancers

**DOI:** 10.18632/oncotarget.9713

**Published:** 2016-05-30

**Authors:** Heping Yan, Vaishali Kapoor, Kim Nguyen, Walter J. Akers, Hua Li, Jalen Scott, Richard Laforest, Buck Rogers, Dinesh Thotala, Dennis Hallahan

**Affiliations:** ^1^ Department of Radiation Oncology, Washington University in St. Louis, St. Louis, Missouri, USA; ^2^ Mallinckrodt Institute of Radiology, Washington University in St. Louis, St. Louis, Missouri, USA; ^3^ Siteman Cancer Center, School of Medicine, Washington University in St. Louis, St. Louis, Missouri, USA

**Keywords:** TIP-1, radiation-inducible, monoclonal antibody, radioimmunotherapy, in vivo imaging

## Abstract

Radiation-inducible neo-antigens are proteins expressed on cancer cell surface after exposure to ionizing radiation (IR). These neo-antigens provide opportunities to specifically target cancers while sparing normal tissues. Tax interacting protein-1 (TIP-1) is induced by irradiation and is translocated to the surface of cancer cells. We have developed a monoclonal antibody, 2C6F3, against TIP-1.

Epitope mapping revealed that 2C6F3 binds to the QPVTAVVQRV epitope of the TIP-1 protein. 2C6F3 binds to the surface of lung cancer (A549, LLC) and glioma (D54, GL261) cell lines. 2C6F3 binds specifically to TIP-1 and ELISA analysis showed that unconjugated 2C6F3 efficiently blocked binding of radiolabeled 2C6F3 to purified TIP-1 protein. To study *in vivo* tumor binding, we injected near infrared (NIR) fluorochrome-conjugated 2C6F3 via tail vein in mice bearing subcutaneous LLC and GL261 heterotopic tumors. The NIR images indicated that 2C6F3 bound specifically to irradiated LLC and GL261 tumors, with little or no binding in un-irradiated tumors.

We also determined the specificity of 2C6F3 to bind tumors *in vivo* using SPECT/CT imaging. 2C6F3 was conjugated with diethylene triamine penta acetic acid (DTPA) chelator and radiolabeled with ^111^Indium (^111^In). SPECT/CT imaging revealed that ^111^In-2C6F3 bound more to the irradiated LLC tumors compared to un-irradiated tumors. Furthermore, injection of DTPA-2C6F3 labeled with the therapeutic radioisotope, ^90^Y, (^90^Y-DTPA-2C6F3) significantly delayed LLC tumor growth. 2C6F3 mediated antibody dependent cell-mediated cytotoxicity (ADCC) and antibody dependent cell-mediated phagocytosis (ADCP) *in vitro*.

In conclusion, the monoclonal antibody 2C6F3 binds specifically to TIP-1 on cancer and radio-immunoconjugated 2C6F3 improves tumor control.

## INTRODUCTION

Tumor-specific targeted drug delivery has the potential to improve the efficacy of cytotoxic agents against tumors while minimizing toxicity to normal tissues. Monoclonal antibodies (mAbs) are one class of agents that can be used for cancer-specific therapeutic and diagnostic applications. mAbs achieve therapeutic effects by (i) antagonizing the receptor signaling or turnover directly (ii) affecting the vasculature or stroma indirectly or (iii) invoking an immune response through activation of complement-dependent cytotoxicity or antibody-dependent cellular toxicity [1, 2]. mAbs have also been conjugated to cytotoxic drugs or radionuclides to directly target cancer cells [3]. Antibodies recognizing biomarkers expressed on the surface of the cancer cells or the tumor vasculature have been successfully used in the clinic. These include bevacizumab that targets VEGF, cetuximab that targets EGFR, pembrolizumab that targets PD1 (T cells), ipilimumab that targets CTLA4 (T cells) and trastuzumab that targets HER2 [4, 5]. Several new surface biomarkers for cancer are being identified and tested pre-clinically for their suitability to be used as targets for immunotherapy.

We previously identified a radiation-inducible neoantigen, Tax interacting protein 1 (TIP-1, also known as Tax1 binding protein 3, (Tax1BP3), that translocates to the cancer cell surface following irradiation [6]. TIP-1 was initially identified as one of the binding partners of the T-cell leukemia viral oncoprotein Tax [6, 7]. A single PSD-95/DlgA/ZO-1 (PDZ) domain (89 amino acids) is the only structural and functional unit in the small protein (total of 124 amino acids in human and mouse), distinguishing TIP-1 from other PDZ proteins. TIP-1 functions as a scaffold for protein complex assembly and is highly conserved across species [8]. TIP-1 plays an important role in cancer, directly or indirectly regulating various signaling pathways that are involved in cancer development and progression. Overexpression of TIP-1 is implicated in cancer cell adhesion, migration, and metastasis [9]. Recently TIP-1 was shown to activate Rho GTPases and facilitate cell migration in glioblastoma [10]. Mouse models of lung cancer were specifically targeted by TIP-1 specific peptide HVGGSSV [11, 12]. Overall, TIP-1′s involvement in many different cancer pathways demonstrates its potential to be explored as a molecular target for cancer therapy.

In the present study, we developed a monoclonal antibody against TIP-1, 2C6F3. We show that it binds to the surface of cancer cells and specifically to radiation-inducible TIP-1. 2C6F3 was conjugated to a radioisotope and was successfully used as a radio-immunoconjugate for the treatment of lung cancer models in mice.

## RESULTS

### The specificity of the 2C6F3 antibody

The specificity of the monoclonal antibody 2C6F3 to TIP-1 was determined by using immunoblotting. We titrated various concentrations of TIP-1 (50, 100,150, 200 and 250 nM) with 25 nM TIP-1 monoclonal antibody 2C6F3 (Supplementary Figure S1A). The TIP-1 monoclonal antibody 2C6F3 detected all concentrations of TIP-1 protein. There was no significant difference in detection of the lowest concentration (50 ng) to the highest concentration (250 ng). We also evaluated the cytosolic induction of TIP-1 protein after irradiation. We irradiated A549, D54 and LLC cells with 3 Gy and immunoblotted for TIP-1 at 24 and 48 h (Supplementary Figure S1B). We found induction of TIP-1 at 24 h and 48 h in irradiated A549, D54 and LLC cells when compared to sham alone. There was a slight increase in TIP-1 induction in all the 3 cell lines at 48 h compared to 24 h.

### Epitope mapping of 2C6F3 and modeling of 2C6F3 epitope on the TIP-1 protein

**Figure 1:**
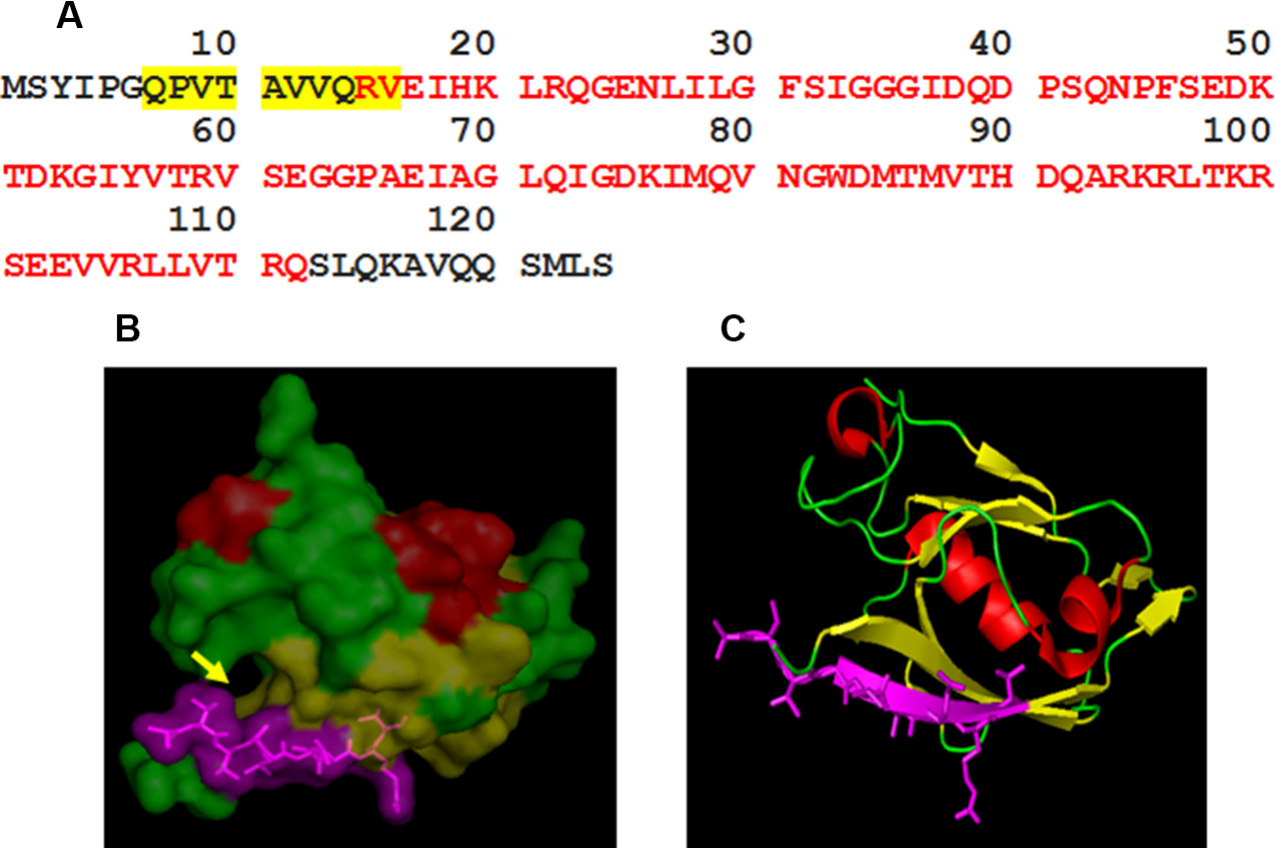
Epitope mapping of 2C6F3 and its location on the TIP-1 protein (**A**) The sequence of the TIP-1 protein with red letters indicating the PDZ domain; the amino acids highlighted in yellow represent the epitope binding sequence of the 2C6F3 monoclonal antibody. (**B**) 3D structure of TIP-1 indicating the 2C6F3 epitope (violet) and the canonical peptide-binding pocket of the PDZ domain (yellow arrow). (**C**) Ribbon model of TIP-1 showing the location of the 2C6F3 Ab epitope (shown in violet).

We developed a monoclonal antibody against mammalian TIP-1, 2C6F3. The mammalian TIP-1 comprised of a single PDZ domain consists of 124 amino acids. We mapped the epitope of the 2C6F3 monoclonal antibody in the TIP-1 protein as described above. Epitope mapping indicated that 2C6F3 specifically bound to amino acids QPVTAVVQRV (Figure 1A; yellow highlighted region). These amino acids are located in the N-terminal region of TIP-1 and just next to the beginning of the PDZ domain indicated in red letters. Previously the high-resolution crystal structure of TIP-1 in complex with the C-terminal peptide of β-catenin has been elucidated [13]. The 3D modeling depicting the canonical peptide-binding pocket of the PDZ domain (yellow arrow) and the 2C6F3 epitope (violet region) are shown in Figure 1B and 1C.

Modeling of QPVTAVVQRV in the three-dimensional crystal structure of TIP-1 using PyMOL software indicated that although 2C6F3 does bind in the N-terminal region of TIP-1, it does not bind in the canonical peptide-binding pocket (Figure 1B and 1C).

### 2C6F3 binds to cancer cell surface and Tip-1 protein *in vitro*

**Figure 2:**
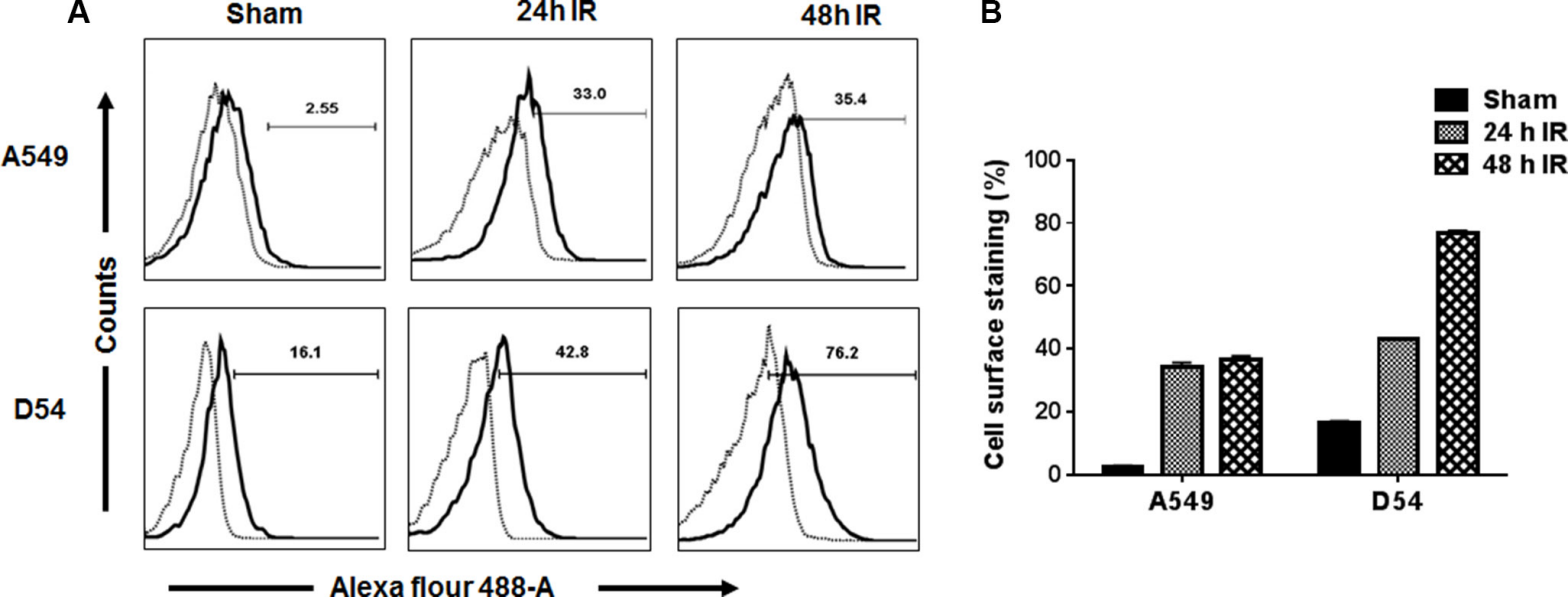
Flow cytometric analysis of the binding of 2C6F3 to the surface of human lung and glioblastoma cells A549 and D54 cells were irradiated with 3 Gy or sham treated. At 24 h and 48 h post-irradiation, cells were collected and stained with 2C6F3 and a secondary antibody labeled with Alexa Flour 488. Shown are the overlay histograms (**A**) and bar graphs (**B**) of cell surface staining in each treatment with SD of three samples.

Binding of 2C6F3 to the surface of cancer cells was evaluated by flow cytometry. A549 (lung cancer) and D54 (glioblastoma) cells were irradiated (3 Gy × 3) to induce the surface expression of TIP-1 and then incubated with 2C6F3 antibody followed by incubation with secondary antibody. A549 and D54 cells treated with the secondary antibody alone were used as negative controls. The flow cytometric analysis indicated that 2C6F3 bound to the cell surface of both A549 and D54 cells (Figure 2A). There was an 11 and 2 fold increase of cell surface associated TIP-1 at 24 h post-IR in A549 and D54 cells respectively. There was no significant increase in expression of surface TIP-1 in A549 at 48 h compared to 24 h. There was a 1.8 fold increase in cell surface expression of TIP-1 in D54 at 48 h when compared to 24 h in the D54 cells (Figure 2B).

### 2C6F3 binds to mouse tumors *in vivo*

**Figure 3:**
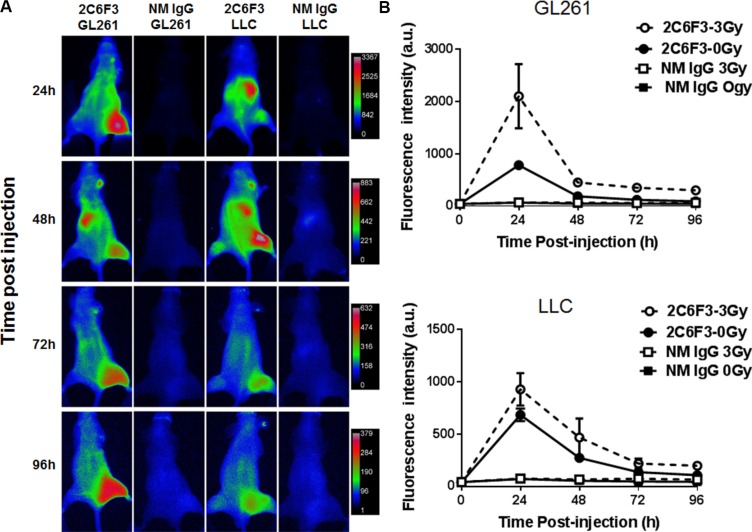
NIR imaging of mice bearing heterotopic tumors (**A**) Mice bearing heterotopic LLC or GL261 tumors were treated with 3 fractions of 3 Gy over the course for 24 hours radiation or sham treated followed by intravenous administration of 50 μg 2C6F3-AF750 or normal mouse IgG-AF750. (**B**) Line graph showing the mean fluorescence intensity of 2C6F3-AF750 or NM IgG-AF750 at various times post injection in irradiated and sham treated tumors.

We next evaluated the efficacy of binding of 2C6F3 to mouse tumor models by optical imaging. We performed near infrared imaging (NIR) of heterotopic tumors of Lewis lung cancer (LLC) and glioblastoma (GL261). Tumors on the right hind limb were irradiated with 3 fractions of 3 Gy over a course of 24 h while the tumor on the left hind limb was used as the sham-irradiated control. The mice were then injected with 50 μg of 2C6F3-Alexa flour 750 (2C6F3-AF750) or 50 μg normal mouse IgG-Alexa flour 750 (IgG-AF750). The NIR imaging was performed at 24 h, 48 h, 72 h and 96 h post antibody injection (Figure 3A). Significant accumulation of the TIP-1 antibody (2C6F3) was observed in irradiated tumors when compared to normal IgG in both LLC and GL261tumors (Figure 3). Mice injected with 2C6F3-AF750 had 2099 a.u. in GL261 and 926 a.u. in LLC tumors at 24 hours following irradiation while un-irradiated tumors had significantly less accumulation of 780 and 683 a.u. in GL261 and LLC tumors respectively (Figure 3B). At 48 h similar accumulation of 451 a.u. (GL261) and 466 a.u (LLC) was seen in irradiated tumors compared to 186 a.u. (GL261) and 271 a.u. (LLC) in un-irradiated tumors. At 72 and 96h there were no significant differences in 2C6F3-AF750 accumulation in irradiated compared to un-irradiated tumors. Low binding of normal mouse IgG-AF750 was observed in both GL261 and LLC tumors at all time points after irradiation or sham treatment (Figure 3B).

### ^125^I-2C6F3 antibody retains TIP-1 specificity and binds specifically to tumors *in vivo*

**Figure 4:**
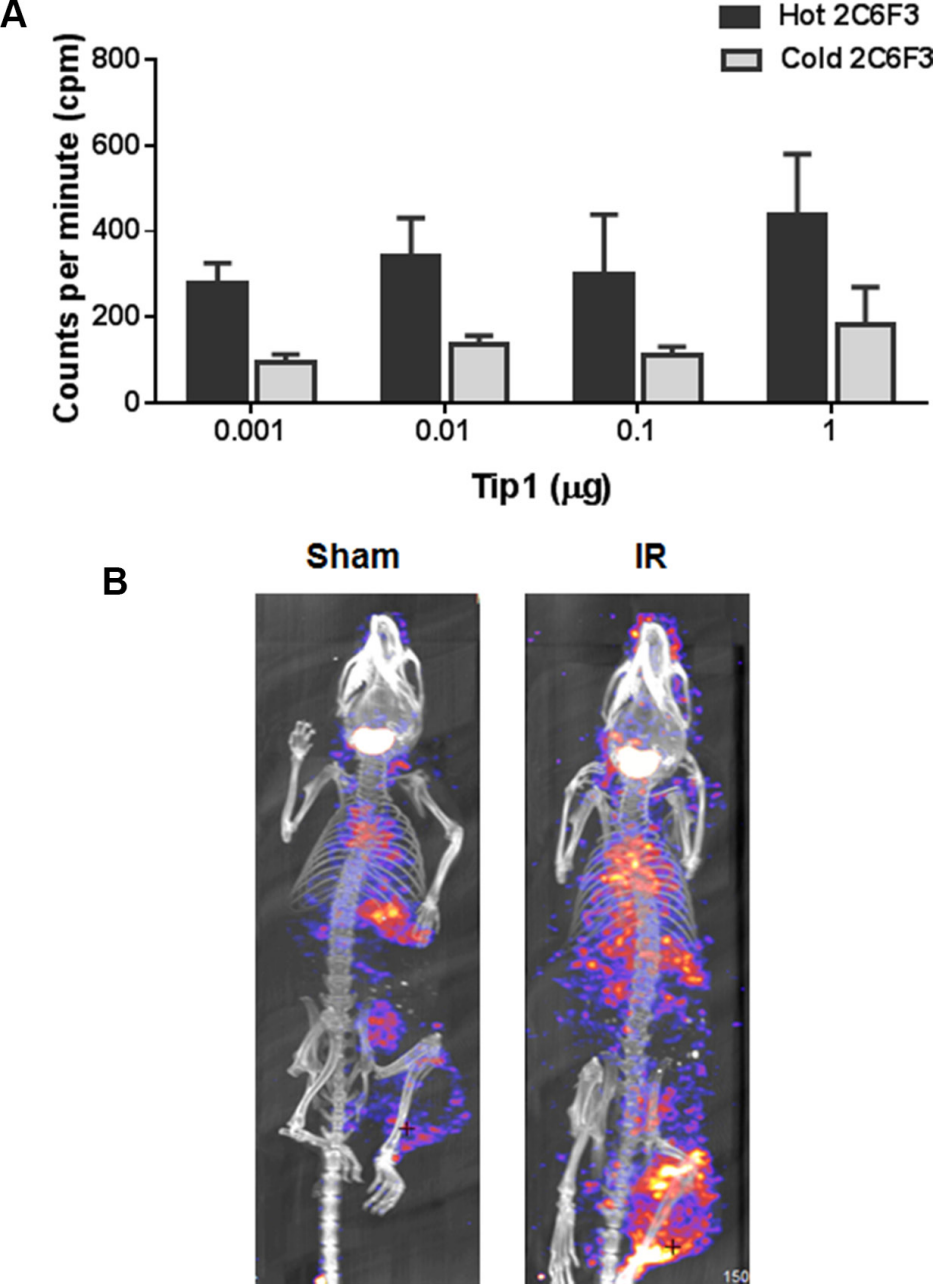
^125^I labeling of 2C6F3 does not affect the efficacy of binding and SPECT imaging (**A**) Bar graph showing ELISA analysis of the competition of unconjugated 2C6F3 with ^125^I-2C6F3 to various concentrations of recombinant TIP-1 protein. (**B**) CT/SPECT imaging of C57/BL6 mice's hind limb LLC tumors injected with ^125^I-2C6F3.

We next wanted to evaluate the efficacy of tumor binding of 2C6F3 antibody after conjugation of ^125^I radio-isotope. To confirm that ^125^I-labeled 2C6F3 retained its specificity to TIP-1 we did competition studies with cold 2C6F3. We found that ^125^I -labeled 2C6F3 retained its binding specificity at as low as 0.001 μg to 1 μg of recombinant TIP-1 protein (Figure 4A). The cold 2C6F3 (200 fold excess) was able to block the binding of ^125^I-labeled 2C6F3 to recombinant TIP-1.

We performed SPECT/CT imaging with^125^I-labeled 2C6F3 on heterotopic LLC tumors in C57BL/6 mice. The tumors were irradiated with 3 fractions 3 Gy over a course of 24 h. The mice were then injected with 120 μCi of ^125^I -labeled 2C6F3 via the tail vein. SPECT/CT imaging was performed at 48 h post injection. The SPECT images revealed that ^125^I -labeled 2C6F3 bound specifically to the irradiated LLC tumors (Figure 4B). Very low or negligible binding of ^125^I -labeled 2C6F3 was observed in sham-irradiated tumors (Figure 4B).

### ^111^In-2C6F3 labeled antibody binds specifically to tumors *in vivo*

**Figure 5:**
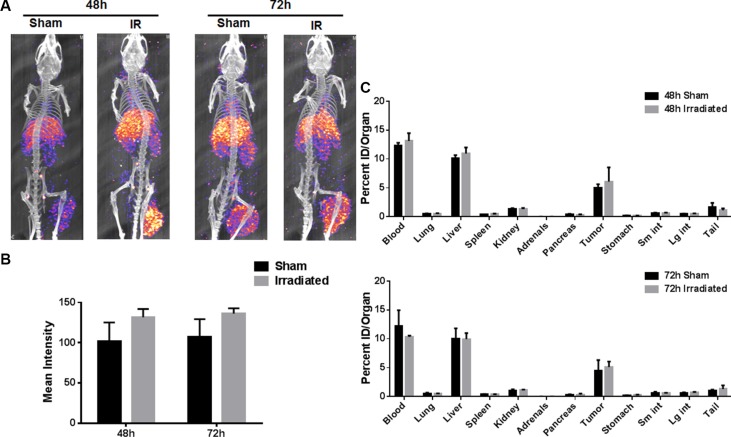
SPECT imaging of the heterotopic LLC lung tumor model with ^111^In labeled 2C6F3 (**A**) SPECT-CT images of mice bearing heterotopic LLC tumors injected with 250 μCi ^111^In-2C6F3. Enhanced tumor binding of the radiolabeled 2C6F3 was observed at 48 h and 72 h post-irradiation (3Gy × 3). (**B**) The mean intensity of the irradiated and sham-irradiated tumors calculated from drawing ROI from SPECT imaging using ImageJ (**C**) Biodistribution of ^111^In labeled 2C6F3 in the irradiated and sham treated heterotopic LLC tumor model. Mice injected with 20 μCi ^111^In-2C6F3 and organs were harvested 48 h and 72 h post-injection. Shown are the biodistribution of ^111^In labeled 2C6F3 in various organs.

Indium DTPA (^111^In-DTPA) is a diagnostic agent and used routinely for cancer imaging. We first conjugated 2C6F3 with DTPA and evaluated the specificity of the DTPA-2C6F3 to TIP-1. We determined that DTPA-2C6F3 retained its specificity for TIP-1 similar to that of 2C6F3 alone (data not shown). We then labeled DTPA-2C6F3 with ^111^In and performed SPECT/CT imaging using heterotopic LLC tumors. Tumors were irradiated with 3 fractions of 3 Gy over the course of 24 hours or sham irradiated. The mice were then injected with 250 μCi of radiolabeled DTPA-2C6F3 via the tail vein. SPECT/CT imaging was performed at 48 and 72 h post injection. The SPECT imaging at 48 h and 72 h post injection revealed that ^111^In-labeled DTPA-2C6F3 bound to both sham and irradiated LLC tumors (Figure 5A). More ^111^In-DTPA-2C6F3 was bound in the irradiated tumors at 48 h and 72 h compared to sham-irradiated control tumors, although not at significant levels (Figure 5B).

The biodistribution of ^111^In-DTPA-2C6F3 was determined at 48 h and 72 h post injection. ^111^In labeled DTPA-2C6F3 accumulated in the blood, liver and tumors in the in tumor-bearing mice (Figure 5C). The radiolabeled 2C6F3 was still in circulation as observed by the blood uptake in the biodistribution data. Labeled 2C6F3 was also observed in the liver as these antibodies were being cleared from the circulation. The levels of the labeled antibody in lung, spleen, kidney, adrenals, pancreas, stomach, and intestine were low.

### Radioimmunotherapy with ^90^Y-DTPA-2C6F3 antibody

**Figure 6:**
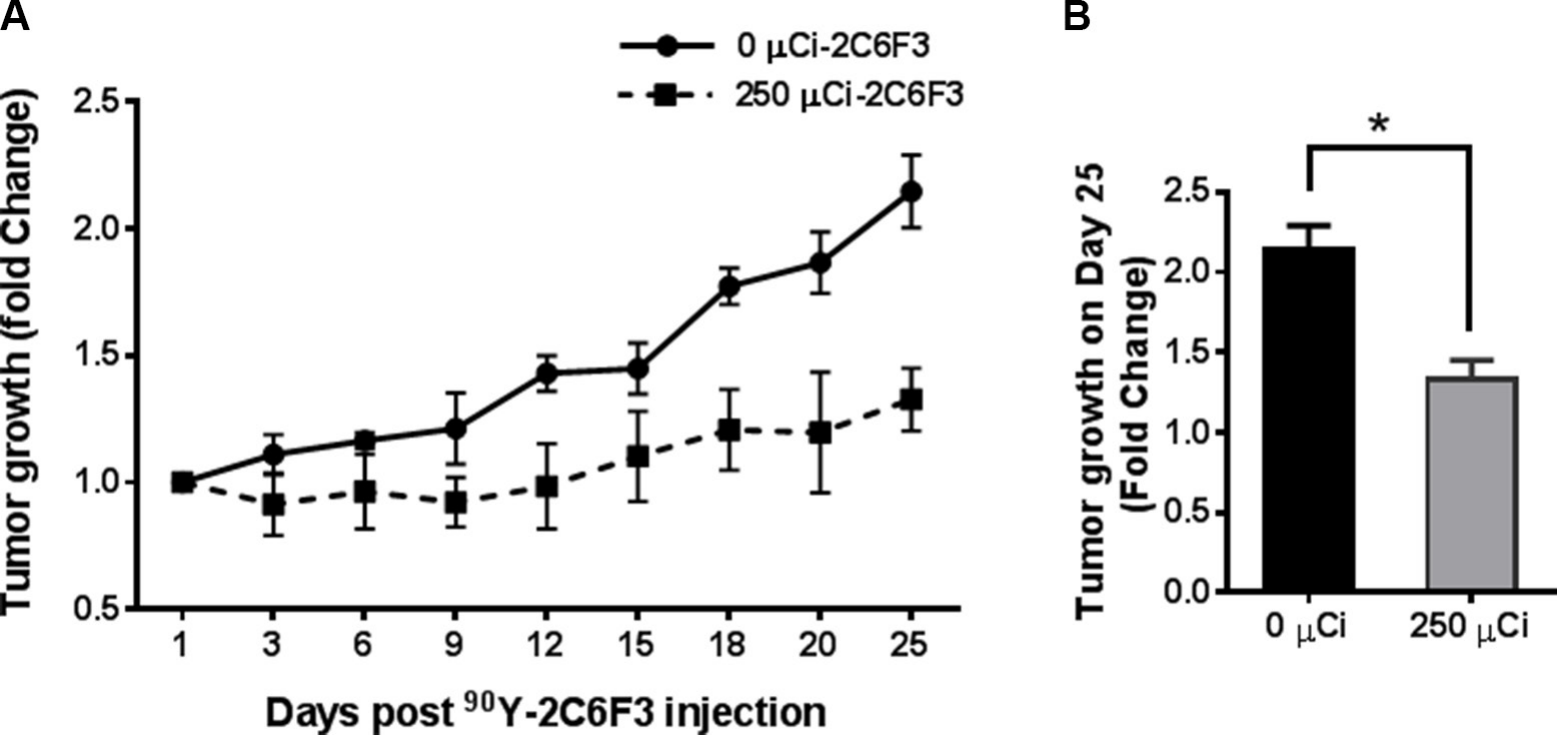
Radioimmunotherapy of heterotopic human lung cancer tumors with yttrium-90 (^90^Y) 2C6F3 antibody (**A**) Athymic nude mice bearing subcutaneous A549 human lung tumors were given a single intravenous injection of 250 μCi of ^90^Y-DTPA-2C6F3 or unlabeled DTPA-2C6F3. Shown are the mean tumors volumes of treatment groups as fold change over time. (**B**) Tumor growth on the 25th day after treatment (*indicates a *P* value < 0.05).

Yttrium-90 (^90^Y) is a therapeutic agent that is used routinely for cancer therapy. The therapeutic efficacy of ^90^Y-DTPA-2C6F3 was evaluated in athymic nude mice bearing heterotopic human A549 lung cancer tumors. A single dose of 250 μCi of ^90^Y-DTPA-2C6F3 was administered via intravenous tail vein injection in a cohort of six mice bearing hind limb tumors. The second cohort of 6 tumor-bearing mice treated with unlabeled DTPA-2C6F3 antibody was used as controls. Mice treated with 250 μCi of ^90^Y-DTPA-2C6F3 showed delayed tumor growth when compared to the cohort that received the unlabeled DTPA-2C6F3 (Figure 6A). There was a significant tumor growth delay on day 25 in mice treated with either 250 μCi of ^90^Y-DTPA-2C6F3 when compared to the control group that received the unlabeled DTPA-2C6F3 (Figure 6B).

### 2C6F3 antibody mediates ADCC and ADCP *in vitro*

**Figure 7:**
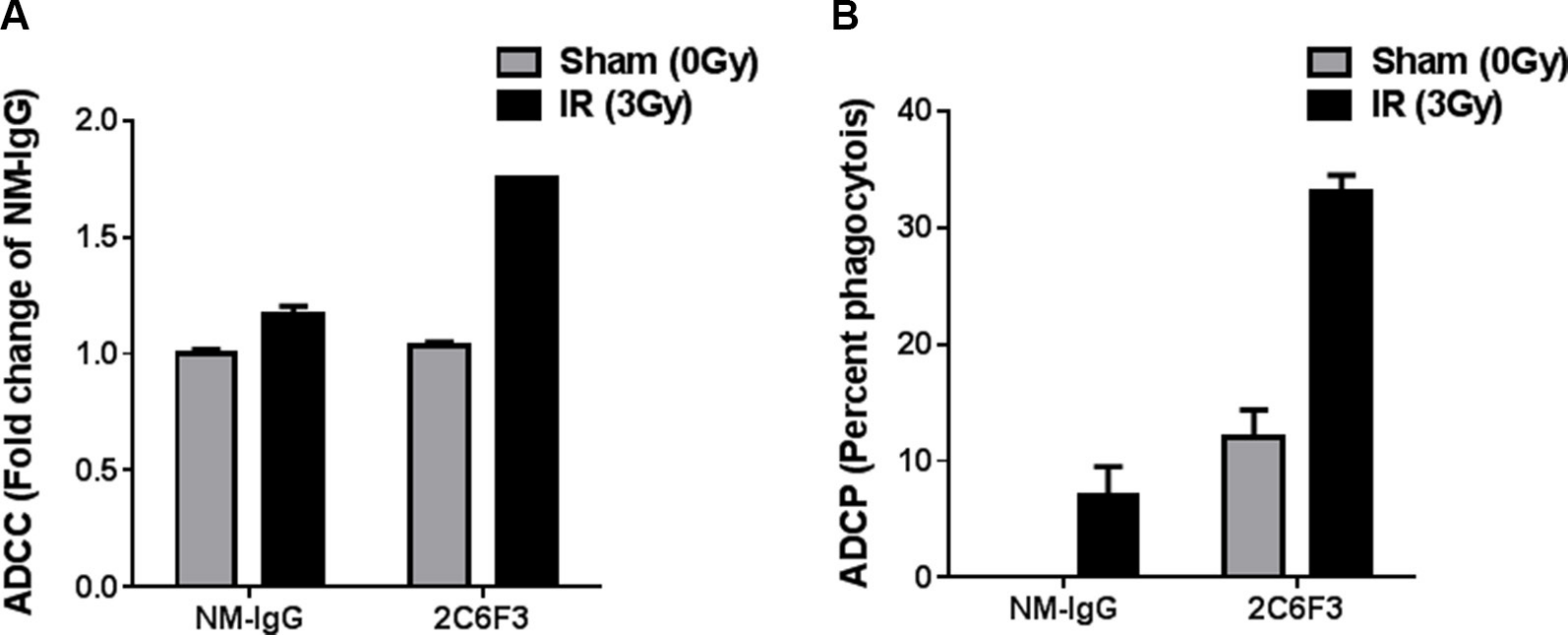
2C6F3 antibody activates ADCC and ADCP *in vitro* (**A**) Antibody-mediated activation of murine NK cells *in vitro* leading to LDH release from LLC cells with or without irradiation. Bar graphs show means with SD of LDH release from triplicates. Data has been normalized after subtracting the values from media alone, tumor cells alone and NK cells alone. (**B**) Antibody-mediated phagocytosis by dendritic cells *in vitro*. Bar graphs show percent phagocytosis by DC from triplicates.

Many antibodies that are being used in the clinic for cancer therapy are known to elicit ADCC and ADCP mechanisms. Therefore, we evaluated the potential of 2C6F3 to mediate immune cell activation *in vitro.* Activation of mouse NK cell-mediated tumor cell lysis was performed by measuring LDH release from tumor cells treated with 2C6F3 antibody. 2C6F3 showed significantly higher killing of irradiated LLC cells (1.7 fold) when compared to irradiated LLC cells treated with NM-IgG (1.1 fold; Figure 7A).

We also evaluated antibody dependent dendritic cell-mediated phagocytosis of LLC. LLC cells treated with 2C6F3 showed enhanced phagocytosis (33%) compared to LLC cells treated with NM-IgG (12% Figure 7B).

## DISCUSSION

Recent success with immunotherapy against various cancers has generated a need for identifying and developing antibodies against specific cancer targets. In this study, we developed and evaluated 2C6F3, a monoclonal antibody that specifically binds to TIP-1 for radioimmunotherapy. TIP-1 is overexpressed in various cancers including lung cancer [14], breast cancer [9] and glioblastoma [10]. TIP-1 consists of a single PDZ domain and has been showed to have various biological functions. These include the stress response [15], cell proliferation [16], cell migration [10] and radioprotection [17]. The levels of TIP-1 expression in patients with glioblastoma has been reported to be a prognostic factor for disease progression [18] and correlated with shorter survival [10]. It has been reported that TIP-1 is induced on the cell surface after radiation in lung cancer and has been implicated as a molecular biomarker for tumor response to radiation [6]. Previously we have demonstrated improved radiation therapy with a peptide that specifically bound to TIP-1 (HVGGSSV) conjugated with nano-albumin-paclitaxel. HVGGSSV-nab-paclitaxel showed tumor-specific binding and enhanced bioavailability of the drug [11].

The monoclonal antibody 2C6F3 developed in this study specifically binds to amino acids QPVTAVVQRV in the N-terminal region of the TIP-1 protein (Figure 1A). Modeling of 2C6F3′s epitope (QPVTAVVQRV) with the crystal structure of TIP-1 indicated that 2C6F3 binds in the N-terminal region of the TIP-1 protein and not in the canonical peptide binding pocket (Figure 1B and 1C). We observed binding of 2C6F3 to the surfaces of lung cancer and glioblastoma cells (Figure 2A and 2B).

Near infrared imaging of tumor-bearing mice injected with 2C6F3-AF750 demonstrated its enhanced binding to irradiated GL261 and LLC tumors when compared to mice injected with normal mouse IgG-AF750 alone at 24 h and 48 h (Figure 3A and 3B). This indicated that the monoclonal antibody against TIP-1 is able to specifically bind lung cancer tumors and glioblastoma. To develop a monoclonal antibody for therapy, monitoring its specificity and distribution is particularly important. The specificity and distribution of antibodies can be monitored noninvasively using PET or SPECT in preclinical mouse tumor models. We labeled 2C6F3 with ^111^In using DTPA as a chelator and imaged heterotopic LLC tumors using SPECT/CT. SPECT/CT imaging at 48 h and 72 h revealed that ^111^In-labeled DTPA-2C6F3 bound to LLC tumors (Figure 5A). Lung cancer expresses TIP1, and irradiation can enhance the expression of TIP-1. The enhanced expression of TIP-1 is dependent upon various factors including cell type, irradiation dose, and time post-irradiation. Although not statistically significant, we observed enhanced binding in irradiated tumors compared to sham-irradiated control tumors. Optimizing the expression of TIP1after irradiation and addition of cohorts of mice could show significant differences between irradiated and sham-irradiated tumors. The biodistribution data supported the SPECT imaging data. Higher uptake of ^111^In- DTPA-2C6F3 was observed in LLC tumors (Figure 5B and 5C).^111^In- DTPA-2C6F3 was also observed in the blood and liver. It has been reported that clearance of radiolabeled antibodies from the blood is dose dependent and is also elevated in patients with high tumor burden [19]. Thus, the higher blood and liver levels could be due to the circulation of ^111^In- DTPA-2C6F3 and tumor burden. Liver uptake of therapeutic antibodies conjugated with drugs including radioisotopes has been reported [20–22]. The enhanced accumulation of 2C6F3 antibody in the liver could be due to its clearance from the liver. This would need to be optimized before translating 2C6F3 into the clinic. It has been suggested that alterations in chelation and linker chemistry may reduce liver uptake of antibodies [23]. Another approach could involve pre-targeting with a streptavidin-conjugated 2C6F3 followed by ^111^In-labeled biotin. This pre-targeting approach is under investigation for several different radio-immunoconjugates used for imaging and therapy [24–27]. Antibodies by themselves may have limited therapeutic efficacy. Therefore, more emphasis is being placed on using antibodies for delivery of toxic agents such as radioisotopes. ^90^Y-conjugated antibodies have been effectively utilized for cancer therapy. For example, Cetuximab that targets the epidermal growth factor conjugated with ^90^Y was effective in controlling tumor growth [28]. In the present study, we targeted TIP-1 using 2C6F3 labeled with ^90^Y to treat human heterotopic lung cancer. 2C6F3 labeled with ^90^Y will irradiate the tumors, and this irradiation is sufficient to enhance expression of TIP-1. Treatment of a human heterotopic lung cancer with ^90^Y- DTPA-2C6F3 led to a significant tumor growth delay (Figure 6).

Antibodies mediate tumor cell killing by several mechanisms which can be broadly classified as direct (receptor blockade, induction of apoptosis, delivery of cytotoxic agents) or indirect (immune-mediated cell killing). Antibodies can engage the immune system and mediate complement-dependent cytotoxicity (CDC), antibody-dependent cellular cytotoxicity (ADCC), antibody-dependent cellular phagocytosis (ADCP) along with regulating T cell function [29, 30]. We observed that 2C6F3 has the ability to mediate both ADCC by mouse NK cells and ADCP by mouse dendritic cells (Figure 7). A similar mechanism of action has been observed for antibodies approved by FDA for cancer treatment, e.g. Trastuzumab (targets ERBB2), Cetuximab (targets EGFR), Rituximab (targets CD20) and Bexxar (targets CD20) [29, 30]. This ability of 2C6F3 to mediate ADCC and ADCP would most likely enhance the therapeutic efficacy of this antibody. However, further evaluation in a mouse tumor model is needed to confirm this potential for indirect effects *in vivo*.

In conclusion, we have demonstrated that TIP-1 is a suitable target for immunotherapy and imaging of cancer; the monoclonal antibody against TIP-1, 2C6F3, can be used to specifically image and treat tumors as a radio-immunoconjugate moiety. Moreover, since TIP-1 is induced by radiation [6, 11] it is an attractive antibody to use in conjunction with radiation therapy.

## MATERIALS AND METHODS

### Cell lines

Human lung adenocarcinoma A549, mouse lung cancer LLC, mouse glioblastoma GL-261 and human glioblastoma D54 cells were cultured in DMEM/F12, human lung cancer H460 cells in RPMI 1640 with 10% fetal bovine serum, penicillin, and streptomycin. They were maintained at 37^ο^C in a 5% CO_2_ incubator.

### Mouse tumor models and irradiation

All animal studies were performed in accordance with the guidelines of the IACUC. Washington University Division of Comparative Medicine approved the animal protocol. Heterotopic tumor models were established in 6 to 8-week-old female athymic nude or C57BL/6 mice (Envigo, USA). Mice were injected subcutaneously in the hind limb with GL-261 (1 × 10^6^cells), A549 (1 × 10^6^ cells) or LLC (5 × 10^5^ cells). Once the tumors were palpable, mice were stratified into treatment groups having similar tumor sizes. Tumors were sham irradiated or irradiated with 3 fractions of 3 Gy (Rad Source RS2000) over a period of 24 hours after anesthetizing the mice using 1–3% isoflurane and shielding the rest of the body with lead.

### Anti-TIP-1 mouse monoclonal antibody production and purification

Six-week-old female BALB/C mice (Envigo, USA) were immunized with recombinant human TIP-1 protein (50 μg) mixed with equal volume of adjuvant (TiterMax Gold adjuvant, Sigma). One month after initial immunization, the mice were boosted with TIP-1 without adjuvant. Three boosters were given at two-week intervals. The mouse having the high immune response to the TIP-1 antigen was chosen as the B cell donor for cell fusion. Spleen cells were fused with mouse myeloma cells (2 × 10^7^ per spleen). The fused cells were plated into 96 well tissue culture plates with HAT selection medium. Fifteen days post fusion hybridoma culture supernatants were assayed. Hybridomas that produced antibodies were sub-cloned to get single-cell clones.

Positive hybridoma clones (e.g. clone 2C6F3) were expanded in serum-free medium. The monoclonal antibodies produced in the serum-free medium were purified by using protein A/G columns.

### Anti-TIP-1 monoclonal antibody 2C6F3 epitope mapping

Twenty peptides that were 10 amino acids long with 4 amino acid overlaps encompassing the 124 amino acids of the human TIP-1 protein were used for epitope mapping. The 96 well ELISA plates were coated with the 20 peptides along with the positive and negative controls. The plates were washed with PBS containing 0.1% Tween20 and blocked with 3% BSA for 45 minutes. The plates were washed and incubated with 5 μg/ml TIP-1 monoclonal antibody 2C6F3 at RT for 2 hours. The plates were washed and incubated with secondary anti-mouse IgG horseradish peroxidase conjugated antibody at RT for 2 hours. After washing, the plates were developed with ABTS substrate and read at 405 nm using an ELISA reader (BioTek Instruments).

### Modeling the 2C6F3 epitope on TIP-1

To identify the location of the epitope on the three-dimensional (3D) structure of TIP-1, a published crystal structure, 3DIW was used [13]. PyMOL software was used to highlight the epitope of 2C6F3 in the TIP-1 3D structure.

### Monitoring TIP-1 surface expression by flow cytometry

The expression of TIP-1 in on the surface lung cancer (LLC, A549, and H460) and Glioma (D54, GL261) cell lines was evaluated using a flow cytometer. Cancer cells were irradiated (3Gy × 3) and harvested 24 h and 48 h post-irradiation. Cells were incubated with 2C6F3 (30 μg/ml) for 1 h on ice followed by incubation for 1 h with anti-mouse Alexa488 labeled secondary antibody. Propidium iodide was added to the cells prior to acquisition for flow cytometric analysis for the exclusion of dead cells. Cells were analyzed using a MACSQuant Analyzer flow cytometer (Miltenyi Biotec) and the data were analyzed with FlowJo software (Tree Star Inc.).

### Optical imaging

The TIP-1 mAb 2C6F3 or the normal mouse IgG (NM-IgG) was labeled with Alexa Fluor 750 as per manufacturer's instructions (Thermo Fisher). Tumors were induced by injecting LLC (0.5 × 10^6^) or GL261 (1 × 10^6^) cells in both the hind limbs of nude mice. The right hind limb tumors were irradiated with 3 fractions of 3 Gy over a course of 24 h while the tumors on the left hind limb were used as the sham-irradiated controls. The tumor-bearing mice were then injected with 50 μg of Alexa Flour-750 labeled 2C6F3 (2C6F3-AF750) or normal mouse IgG (IgG-AF750) via the tail vein. For optical imaging, the mice were anesthetized with 2% isoflurane and imaged using the *In-Vivo* Multispectral Imaging System (Bruker Biospin). Fluorescence was detected using 730 nm excitation and 790 nm emission filters with 60 s acquisition time, F-stop 2.4, and 2 × 2 binning. ROI analysis was performed using NIH ImageJ image processing software and mean fluorescence intensity values reported as arbitrary units (a.u.).

### ^125^I labeling and binding assay

2C6F3 (1.0 mg) was mixed with ^125^I (5.0 mCi) in an Iodogen-coated glass tube. The mixture was incubated at room temperature for 15 min and then purified by passing through a PD-10 size-exclusion column. The purity of the ^125^I labeled 2C6F3 was determined using radio-thin layer chromatography (radio-TLC). For binding assays, the TLC plate was coated with 0.001, 0.01, 0.1 and 1 μg of recombinant TIP-1 followed by the addition of 0.1 μg of ^125^I labeled 2C6F3 (0.3 μCi/μg) and incubated for 1 h at room temperature. For blocking assays, the plate was coated with 0.001, 0.01, 0.1 and 1 μg of recombinant TIP-1 and 20 μg of cold 2C6F3 antibody were added per well and incubated for 1 h at room temperature. To this 0.1 μg of ^125^I labeled 2C6F3 (0.3μCi/μg) was added per well and incubated for 1 h at room temperature. The binding efficiency was measured by monitoring the ^125^I activity using a scintillation counter.

### Conjugation of DTPA to 2C6F3 antibody

Diethylene triamine penta acetic acid (DTPA)-NCS was added to 2C6F3 in DTPA to antibody ratio of 10:1 in 0.1 MNa_2_CO_3_ (pH~9) buffer. The reaction mixture was incubated at 37°C for 1h with continuous mixing. The unconjugated DTPA was removed from the conjugated antibody using a 40 kDa Zeba Spin desalting column (Thermo Fisher). The DTPA-conjugated antibody was stored at 4°C in PBS.

### Radiolabeling of DTPA-conjugated 2C6F3

^111^InCl_3_ (370MBq ml^−1^ in 0.5M Hcl, pH1.5) was obtained from Mallinckrodt Pharmaceuticals. An equal volume of ammonium acetate (0.1 M; pH 8.1) was added to ^111^InCl_3_ (pH 1.5) to attain a pH of 5.5. DTPA-2C6F3 was added at specific activity of 1mCi ^111^InCl_3_ per mg of antibody. The mixture was incubated at 37^°^C for 1h on thermomixer. Labeling efficiency was determined using instant thin-layer chromatography (ITLC) using 50mM DTPA. If the detected labeling efficiency was less than 95%, then the mixture was further purified with spin desalting column (40 kDa) to yield more than 95% purity. The ^111^In labeled DTPA-2C6F3 was used for SPECT imaging and biodistribution study.

### Small animal SPECT/CT imaging

Mice bearing heterotopic tumors were injected intravenously either with ^125^I labeled 2C6F3 or ^111^In-DTPA-2C6F3. Whole body SPECT images were obtained at 48 and 72 h post injection (p.i.) using a SPECT/CT imager (Bioscan Inc., Washington, DC, USA) fitted with 2 mm pinhole collimators in the helical scanning mode. Mice were placed in prone position and scanned under anesthesia (0.5 L/min 1.5% isoflurane in air). A 45-keV helical CT scan was performed first and then the SPECT acquisition was performed at 24 projections with 60 s per projection. Tomographic data were reconstructed iteratively with InVivoScope and HiSPECT software for CT and SPECT, respectively. The binding intensity of 2C6F3 in irradiated and sham-irradiated tumors was evaluated using ImageJ software by drawing the regions of interest (ROI) and depicted as mean intensity.

### Biodistribution studies

Mice (*n* = 3 per group) bearing heterotopic LLC tumors were injected intravenously with 20 μCi ^111^In-DTPA-2C6F3. The labeled 2C6F3 in blood, lung liver, spleen, kidney, adrenals, pancreas, stomach, small and large intestine, tumor and tail was determined. The mice were sacrificed and the organs of interest were dissected/collected, weighed, and counted in a gamma counter along with a standard of the injected activity to allow calculation of the injected dose per organ (% ID/organ).

### Tumor growth delay with yttrium-90 (^90^Y)-DTPA-2C6F3 antibody

The growth delay of heterotopic A549 (human lung cancer) tumors with ^90^Y-DTPA-2C6F3 was evaluated in athymic nude mice. Cohorts of six A549 tumor-bearing mice (average tumor size 450 ± 50 mm^3^ and average mouse weight 31.24 ± 1.23 g) were treated with a single intravenous injection of 250 μCi of ^90^Y- DTPA-2C6F3. A cohort of 6 mice treated with 200 μg of unlabeled DTPA-2C6F3 was used as controls. Tumor measurements and animal weights were recorded every 2–3 days and tumor volumes were calculated as fold change from the size of the tumor at the beginning of the therapy.

### Antibody dependent cell-mediated cytotoxicity (ADCC)

Murine natural killer cells (NK cells) were isolated from spleen by using a MagCellect mouse NK cell Isolation Kit (R&D Systems, Inc.) as per manufacturer's protocol. LLC cells were cultured in 96 well plate (5000 cells/well). Following incubation for 40 hours, the cells were irradiated with 3 Gy or sham (0 Gy) and allowed to incubate for another 4 hours. Tumor cells were then treated with 10 μg /ml of mouse anti TIP1 (2C6F3) or normal mouse IgG (NM-IgG) antibody and incubated for additional 2 hours. The murine NK cells were added to the target tumor cells at a ratio of 5:1 (effector cells: target cells) and allowed to incubate for 16h. Following incubation, the culture supernatants were collected and analyzed for Lactate dehydrogenase (LDH) using an LDH cytotoxicity detection kit (Roche). The amount of LDH released was monitored as absorbance at 490 nM.

### Antibody dependent cell-mediated phagocytosis (ADCP)

LLC cells were irradiated (3 Gy), stained with DiI dye (Thermo Scientific) and then treated with 2C6F3 antibody. Mouse dendritic cells (DC) were isolated and grown in culture media containing rmGM-CSF, rmTNFα and rmCD40 ligand for maturation. These mature DC were then co-cultured with LLC cells pretreated with 2C6F3 (target) at a ratio of 1:1 for 24 hrs. The co-cultured cells were then fixed and counterstained with DAPI. The number of phagocytized tumor cells was quantified by counting the number of DC that engulfed DiI-labeled cancer cells in 50 high power fields using fluorescent microscope (Olympus).

### Statistical analyses

The mean and standard error of the mean (SEM) of each treatment group were calculated based on at least three replicates for all experiments. Experiments were repeated at least three times. All pairwise comparisons, including calculation of *P* values, were done using the Student's *t*-test. The significance of the difference in tumor growth rates in the treatment and control groups was determined using Wilcoxon one-sided tests.

## SUPPLEMENTARY MATERIALS


