# Supplementary material for: Anti-tax interacting protein-1 (TIP-1) monoclonal antibody targets human cancers
Source: Oncotarget. 2016 May 30;7(28):43352–62. doi: 10.18632/oncotarget.9713 (PMC5190028; doi:10.18632/oncotarget.9713)
Supplement: Supplementary file 1 [file oncotarget-07-43352-s001.pdf]

Anti-tax interacting protein-1 (TIP-1) monoclonal antibody targets human cancers

Supplementary Materials

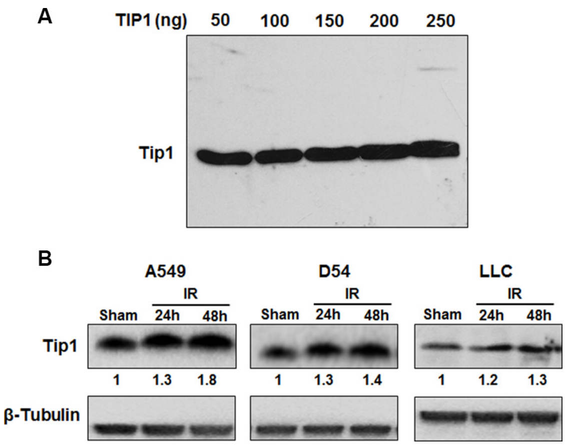

Supplementary Figure S1: Specificity of the 2C6F3 antibody. (**A**) 2C6F3 specifically binds to TIP-1. Various concentrations of recombinant TIP-1 as indicated was immunoblotted with 25 ng TIP-1monoclonal antibody 2C6F3. (**B**) A549, D54, and LLC cells were irradiated with 3 Gy. At 24 h and 48 h after irradiation, total cellular proteins were analyzed for levels of TIP-1 expression. The blots were also immunoblotted with a β-tubulin antibody for loading control.
